# Developmental Model of Depression Applied to Prenatal Depression: Role of Present and Past Life Events, Past Emotional Disorders and Pregnancy Stress

**DOI:** 10.1371/journal.pone.0012942

**Published:** 2010-09-22

**Authors:** Jacques Dayan, Christian Creveuil, Michel Dreyfus, Michel Herlicoviez, Jean-Marc Baleyte, Veronica O'Keane

**Affiliations:** 1 Department of Child Psychiatry / INSERM U923, Caen University Hospital, Caen, France; 2 Biostatistics and Clinical Research Unit, Caen University Hospital, Caen, France; 3 Department of Obstetrics and Gynecology, Caen University Hospital, Caen, France; 4 Department of Psychiatry, Jonathan Swift Unit, St James's Hospital, Dublin, Ireland; RAND Corporation, United States of America

## Abstract

**Background:**

Several risk factors for depression during pregnancy have already been established. However, very few studies have conducted a multivariate analysis incorporating both the major predictors of depression in women, in accordance with comprehensive developmental models of depression, and specific stressors associated with the biological and psychosocial state of the mother-to-be.

**Methodology/Principal Findings:**

We used a cross-sectional cohort design to analyze the associations between prenatal depression and potential risk factors. 693 French-speaking women with singleton pregnancies at 20–28 weeks' gestation were consecutively recruited at Caen University Hospital. Fifty women with missing values were subsequently excluded from the analysis. Depressive symptoms were assessed on the Edinburgh Postnatal Depression Scale. Risk factors were either extracted from the computerized obstetric records or assessed by means of self-administered questionnaires. The associations between prenatal depression and the potential risk factors were assessed using log-binomial regression models to obtain a direct estimate of relative risk (RR). The following factors were found to be significant in the multivariate analysis: level of education (*p*<0.001), past psychiatric history (adjusted RR = 1.8, 95% confidence interval (CI): 1.1;2.8, *p* = 0.014), stress related to the health and viability of the fetus (adjusted RR = 2.6, 95% CI: 1.6;4.1, *p*<0.001), and stress related to severe marital conflicts (adjusted RR = 2.4, 95% CI: 1.5;3.9, *p*<0.001) or to serious difficulties at work (adjusted RR = 1.6, 95% CI :1.04;2.4, *p* = 0.031). An association was also found with the previous delivery of a child with a major or minor birth defect (adjusted RR = 2.0, 95% CI: 1.04;4.0, *p* = 0.038). Univariate analyses revealed a strong association with childhood adversity (parental rejection: RR = 1.8, 95% CI: 1.2;2.8, *p* = 0.0055 and family secrets: RR = 2.0, 95% CI: 1.2;3.1, *p* = 0.0046) and with lack of partner support (RR = 0.50, 95% CI: 0.30;0.84, *p* = 0.0086).

**Conclusions/Significance:**

Our study identifies several risk factors that could easily be assessed in clinical practice. It draws attention to the impact of previously delivering a child with a birth defect. The association with childhood adversity warrants further study.

## Introduction

Studies of the psychosocial correlates of prenatal depression have dramatically increased over the last decade. This recent interest is justified by new evidence of the high prevalence of the disorder and its potentially severe outcome. Based on the results of two recent meta-analyses, the mean prevalence rate of prenatal depression has been estimated to be approximately 12%, although prevalence may vary greatly according to location, mode of assessment and socioeconomic conditions [1]–[4]. A large percentage of women who are depressed during pregnancy remain depressed after birth [5], resulting in an increased risk of insecure attachment and impaired development of the child. As described in a review [6], recent prospective cohort studies have reported significant associations between prenatal depression and several adverse obstetric, fetal and neonatal outcomes, including preterm labor and preterm birth [7]–[10], preeclampsia [11], [12], epidural analgesia, Cesarean section, and admission of the newborn to a neonatal care unit [13]–[15]. Modified fetal cardiac and motor reactivity [16]–[18], and restricted fetal growth [19] have also been consistently linked to a higher rate of prenatal depression. Newborns may also be affected in their emotional behavior and communication [20], although their lack of expressivity may also result from pharmacological treatment of prenatal depression [21]–[24].

Depression in the general population can be brought on by many factors, including genetic influences, childhood risk factors, personality traits, prior onset of anxiety or depressive disorders, exposure to traumatic events and major adversity, low social support, substance misuse, marital conflicts and recent stressful life events and difficulties. Recent studies have confirmed that some of these risks also pertain to prenatal depression. They include personality traits (high neuroticism score, negative cognitive attributional style, low self-esteem) [5], [25], a past psychiatric history [5], [26]–[28], low income and low educational attainment [29]–[31], unemployment [31], previous pregnancy losses, be they miscarriages or pregnancy terminations [32], adverse childhood events, in particular childhood sexual abuse [5], [33], current stressors during pregnancy or in the months beforehand [5], [26], [29], [32], poor maternal physical health [34], low social support and conflict with partner [35].

However this bid to improve our knowledge about risk factors for prenatal depression has been held back by methodological limitations, including the selection of populations of highly socioeconomically disadvantaged women, the use of nonvalidated instruments to assess depressive symptoms, and a lack of data about the course of the pregnancy. More importantly, the etiological complexity of depression requires the assessment of a broad array of interacting variables [36]–[38]. Kendler et al. proposed a comprehensive developmental model of depression in women [37], [38] which assumes that the triggering of a depressive disorder depends on several domains of risk factors that appear successively across the lifespan and interact. In this model, childhood adversity and early life stresses make individuals more vulnerable to later negative events, increasing the risk of subsequent depression. Numerous interacting factors may contribute to this vulnerability, some of which are biological and related to stress responses. There is accumulating evidence from animal and human studies that the hypothalamic-pituitary-adrenal (HPA) axis can become sensitized if individuals are exposed to excessive stressors in early life, leading to an increase in these stress hormones in response to subsequent stressful events [39]. During pregnancy, stress may also heighten the secretion of placental corticotropin-releasing factor (CRF) [40], although its role in prenatal depression is still poorly understood [8].

The purpose of our study was to assess major risk factor domains, including stressors specific to pregnancy, in a population of women receiving regular antenatal care, assessed on a depression scale validated during pregnancy, in order to gain a better understanding of the etiology of prenatal depression.

## Materials and Methods

### Ethics Statement

The analysis presented in this paper is part of a larger study of psychological factors during pregnancy. The study protocol was reviewed and approved by the Lower Normandy Ethics Committee (Consultative Committee for the Protection of Persons participating in Biomedical Research, CCPPRB). Written informed consent was obtained from all participants involved in the study.

### Objective

The objective was to identify the risk factors for depression in mothers-to-be, examining general predictors for depression as well as factors that are particularly relevant during pregnancy. We examined sociodemographic, obstetric, medical and psychosocial (stressful life events, childhood adversity, past psychiatric history, social support) risk factors.

### Participants

Women were consecutively recruited between October 1997 and September 1998, during a prenatal visit to the Department of Obstetrics and Gynecology of Caen University Hospital (France). Enrolment took place within the context of a prospective study of psychological factors during pregnancy. Two papers, focusing on the role of prenatal depression in the onset of preterm labor and preterm birth, have already been published [8], [9]. We used the data collected from the same cohort of women to investigate risk factors for depression during pregnancy.

To be eligible for inclusion in the study, women had to be French-speaking, between 18 and 45 years of age, and between 20 and 28 weeks' gestation. Exclusion criteria were multiple gestation, placenta previa, cervical cerclage and delivery at another hospital. A total of 693 women met the requirements of the protocol.

### Data Collection

Sociodemographic and biomedical characteristics were extracted from the computerized obstetric records. Sociodemographic characteristics included age, marital status, ethnicity, level of education, employment status and smoking habits during pregnancy. Obstetric and clinical characteristics included parity, pre-pregnancy body mass index (BMI; weight (kg)/height (m^2^)), history of pregnancy interruption (miscarriage, ectopic pregnancy, elective or therapeutic abortion), history of preterm birth, history of a child with a major or minor birth defect, chronic medical condition (hepatic, endocrine, neurological, immunological or cardiac), hospitalization during first or second trimester, and complications of the current pregnancy. Pregnancy complications included vaginal bleeding, urinary tract infection, cervical and/or vaginal infection, gestational hypertension (≥140 and/or 90 mmHg), anemia, small or large for gestational age fetus and amniotic fluid anomalies.

Self-administered questionnaires were used for the psychosocial assessment. Women were encouraged to participate by a psychologist and a midwife, who could also provide help with the questionnaires. Several categories of factors were explored: stressful life events, childhood adversity, past psychiatric history and social support. After reviewing the literature on the association between stressful life events and depression in women, we identified a list of seven such events: serious difficulties at work, serious housing problems, major financial problems, severe marital conflicts, serious illness or injury (self or others), physical or sexual assault and legal problems. For each event, women were asked if they had experienced it within the previous 12 months and, if they had, to rate the frequency of rehearsal, on a four-point Likert-type scale (1:“never or hardly ever”, 2: “from time to time”, 3: “often”, 4: “always or almost always”). Events were considered stressful in the case of a high rehearsal frequency (3 or 4 on the Likert scale). An additional stressor, specific to the pregnancy period, was also evaluated: stress related to the fetus. This was deemed to be present if the pregnant woman had been informed by the obstetric team of an ongoing risk or an uncertainty about the health of the fetus and had, for this reason, been referred for a specific consultation or investigation (fetal anatomy scan, amniocentesis, pre-admission testing prior to hospitalization). Childhood adversity was assessed through the following five binary (yes/no) events: physical abuse, sexual abuse, institutional or foster family placement, feeling rejected by at least one of the parents (parental rejection) and family secrets. Past psychiatric history was evaluated by asking the women if they had already had any contact with psychiatric or psychological services. Finally, the social support evaluation consisted of five questions on partner support, mother support, father support, in-law support and other forms of social support.

The outcome variable (occurrence of depressive symptoms) was assessed on the Edinburgh Postnatal Depression Scale (EPDS), a 10-item screening tool that provides an indication of symptom severity. Items are rated on a four-point Likert-type scale ranging from 0 to 3. The EPDS is the only rating scale for depression that has been validated in both the antenatal [41] and postnatal periods [42]. The French version of the scale has also been validated both during pregnancy [43] and postpartum [44]. Reliability of the scale in the current study was found to be good (Cronbach's α = 0.86). A score greater than 14 was deemed to indicate the presence of prenatal depression. This high cut-off value allows for good detection of both major and minor depression in childbearing women. For major depression, sensitivity at higher cut-off points (>14) is almost 100% and specificity is 94% [41].

### Statistical Analyses

Fifty women (7.2%) were excluded from the analysis because they left at least one of the questionnaires completely blank. The sociodemographic, obstetric and clinical characteristics of the nonresponders were similar to those of the responders, except for employment status (more unemployed women) and smoking habits (more heavy smokers). For 43 women in the remaining sample, a single item was missing from the questionnaires; and for 7 women, 2–4 items were missing. To avoid excluding these 50 additional women, any missing values were replaced by the median values for those items. The final sample consisted of 643 women.

The associations between prenatal depression and each of the potential risk factors were assessed using univariate log-binomial regression models to gain a direct estimate of relative risks (RRs), as odds ratios overestimate RRs when the outcome is not rare. Crude RRs with a 95% confidence interval (CI) were computed. A forward multiple log-binomial regression analysis was then applied to all the factors found to be related to the outcome at the *p*<0.20 level in the univariate analyses. Adjusted RRs with 95% confidence limits were computed. Statistical significance was defined as *p*<0.05. Data were analyzed with SPSS software, version 15.0 (SPSS for Windows, Chicago, IL: SPSS Inc., 2006).

## Results

The participants' sociodemographic, obstetric and clinical characteristics are shown in Tables 1 and 2. Mean maternal age was 28.5 years (standard deviation [*SD*]: 5.5; range: 18–45). Women were mainly European in origin and not single; 63% of them had a vocational qualification or had attended higher education, and more than 62% were in some form of employment. Gestational age at enrolment ranged from 20 to 28 completed weeks, with a mean of 23 weeks (*SD*: 2.2). Parity ranged from 0 to 7, with a mean of 1.1. Frequencies of obstetric risk factors were usually below 10%, except for history of pregnancy interruption (39.3%) and cervical and/or vaginal infection (22.6%). Psychosocial characteristics are shown in Table 3. The mean depression score was 7.3 (*SD*: 5.6; range: 0–28) and 74 women (11.5%) had a high depression score (>14).

**Table 1:** Sociodemographic characteristics of study population (*n* = 643).

Characteristics		*n*	%
Marital status	Living alone	71	11.0
	Married or cohabiting	572	89.0
Ethnicity	Europe	607	94.4
	Other	36	5.6
Level of education	Primary school	50	7.8
	Secondary school	188	29.2
	Vocational qualification	234	36.4
	Higher education	171	26.6
Employment status	Unemployed	243	37.8
	Poorly paid job	264	41.1
	Moderately or highly paid job	136	21.2
Smoking habits during pregnancy	Nonsmoking	420	65.3
	1–9 cigarettes per day	144	22.4
	10 cigarettes or more per day	79	12.3

**Table 2:** Obstetric and clinical characteristics of study population (*n* = 643).

Characteristics	*n*	%
History of pregnancy interruption	253	39.3
History of preterm birth	36	5.6
History of child with major/minor birth defect	38	5.9
Chronic disease	72	11.2
Hospitalization during 1^st^ trimester	17	2.6
Hospitalization during 2^nd^ trimester	49	7.6
Vaginal bleeding	65	10.1
Urinary tract infection	70	10.9
Cervical and/or vaginal infection	145	22.6
Gestational hypertension	40	6.2
Anemia	58	9.0
Small or large for gestational age fetus	40	6.2
Amniotic fluid anomalies	26	4.0

**Table 3:** Psychosocial characteristics of study population (*n* = 643).

Characteristics	*n*	%
Stress related to the fetus	84	13.1
Stress related to serious difficulties at work	241	37.5
Stress related to serious housing problems	139	21.6
Stress related to major financial problems	181	28.1
Stress related to severe marital conflicts	52	8.1
Stress related to serious illness or injury (self or others)	138	21.5
Stress related to physical or sexual assault	18	2.8
Stress related to legal problems	11	1.7
Institutional or family placement during childhood	68	10.6
Physical abuse during childhood	68	10.6
Sexual abuse during childhood	45	7.0
Parental rejection during childhood	146	22.7
Family secrets during childhood	102	15.9
Previous psychiatric or psychological consultation	122	19.0
Partner support	570	88.6
Mother support	566	88.0
Father support	484	75.3
In-law support	462	71.9
Other social support	543	84.4

The results of the univariate analyses are set out in Tables 4, 5 and 6. Regarding sociodemographic characteristics (Table 4), we observed a strong association with low levels of education, and a weaker one with high numbers of cigarettes smoked per day. There was a trend toward an association between depression and employment status. Four obstetric factors appeared to be moderately associated with prenatal depression: parity, pre-pregnancy BMI, hospitalization during 2^nd^ trimester and a history of having previously had a child with a major or minor birth defect (Table 5). There were no significant associations between depression and identified obstetric complications in the current pregnancy, but the urinary tract infection, cervical and/or vaginal infection and anemia variables were kept for the multivariate analysis, as the associated *p*-values were below 0.20. Generally, stressful life events were strongly associated with depression (Table 6), apart from stress related to major financial problems (RR = 1.3, *p* = 0.25), physical/sexual assault (RR = 0.97, *p* = 0.96) or legal problems (RR = 0.78, *p* = 0.80). The prevalence of the last two factors was, however, very low, with just 18 and 11 cases, respectively. A strong association was also observed with stress related to the fetus, with an RR close to 2.5 (*p*<0.001). Regarding adverse childhood events, parental rejection and family secrets were clearly significant, with RRs close to 2. The association was weaker and nonsignificant for institutional or family placement. As expected, a strong link emerged with past psychiatric history (RR = 2.6, *p*<0.001). Finally, the influence of social support was significant for partner support (RR = 0.50, *p* = 0.0086), but not for any other form (*p*-values around 0.10).

**Table 4:** Crude relative risks of prenatal depression for sociodemographic characteristics (*n* = 643).

Variables*		% depression	RR	(95% CI)	*p*
Level of education	Primary	20.0	2.8	(1.3 ; 6.2)	
	Secondary	17.6	2.5	(1.3 ; 4.7)	0.0015
	Vocational qualification	8.1	1.2	(0.58 ; 2.3)	
	Higher education	7.0	1		
Employment status	Unemployed	14.8	2.0	(1.03 ; 3.9)	
	Poorly paid	10.6	1.4	(0.72 ; 2.9)	0.084
	Moderately / highly paid	7.4	1		
Smoking habits during pregnancy	Nonsmoking	9.0	1		
	1–9 cig. per day	15.3	1.7	(1.04 ; 2.8)	0.024
	≥10 cig. per day	17.7	2.0	(1.1 ; 3.4)	

*Variables associated with prenatal depression at the *p*<0.20 level.

**Table 5:** Crude relative risks of prenatal depression for obstetric and clinical characteristics (*n* = 643).

Variables*		% depression	RR	(95% CI)	*p*
Parity	0	9.6	1		
	1	8.6	0.90	(0.50 ; 1.6)	0.021
	2	16.4	1.7	(0.97 ; 3.0)	
	≥3	19.7	2.1	(1.1 ; 3.8)	
Prepregnancy body mass index	<18.5	21.2	2.4	(1.2 ; 4.7)	
	18.5–25	10.9	1.2	(0.68 ; 2.2)	0.024
	≥25	9.0	1		
History of child with major/minor birth defect	No	10.9	1		
	Yes	21.1	1.9	(1.00 ; 3.7)	0.050
Hospitalization during 2^nd^ trimester	No	10.8	1		
	Yes	20.4	1.9	(1.04 ; 3.4)	0.037
Urinary tract infection	No	10.8	1		
	Yes	17.1	1.6	(0.90 ; 2.8)	0.11
Cervical and/or vaginal infection	No	10.6	1		
	Yes	14.5	1.4	(0.85 ; 2.2)	0.20
Anemia	No	10.9	1		
	Yes	17.2	1.6	(0.86 ; 2.9)	0.14

*Variables associated with prenatal depression at the *p*<0.20 level.

**Table 6:** Crude relative risks of prenatal depression for psychological characteristics (*n* = 643).

Variables*		% depression	RR	(95% CI)	*p*
Stress related to the fetus	No	9.5	1		
	Yes	25.0	2.6	(1.7 ; 4.1)	<0.001
Stress related to serious difficulties at work	No	9.0	1		
	Yes	15.8	1.8	(1.1 ; 2.7)	0.0094
Stress related to serious housing problems	No	10.1	1		
	Yes	16.5	1.6	(1.04 ; 2.6)	0.034
Stress related to severe marital conflicts	No	9.5	1		
	Yes	34.6	3.7	(2.3 ; 5.7)	<0.001
Stress related to serious illness or injury	No	10.1	1		
	Yes	16.7	1.7	(1.05 ; 2.6)	0.031
Placement during childhood	No	10.8	1		
	Yes	17.6	1.6	(0.93 ; 2.9)	0.087
Parental rejection during childhood	No	9.7	1		
	Yes	17.8	1.8	(1.2 ; 2.8)	0.0055
Family secrets during childhood	No	10.0	1		
	Yes	19.6	2.0	(1.2 ; 3.1)	0.0046
Psychiatric or psychological history	No	8.8	1		
	Yes	23.0	2.6	(1.7 ; 4.0)	<0.001
Partner support	No	20.5	1		
	Yes	10.4	0.50	(0.30 ; 0.84)	0.0086
Mother support	No	16.9	1		
	Yes	10.8	0.64	(0.37 ; 1.1)	0.11
Father support	No	15.1	1		
	Yes	10.3	0.68	(0.44 ; 1.1)	0.10

*Variables associated with prenatal depression at the *p*<0.20 level.

As observed in Table 7, only six factors still appeared to be significant after the multivariate analysis. There continued to be a clear dose-effect relationship for level of education, but the only obstetric factor left in the model was a history of having a child with a birth defect, with an adjusted risk equal to 2. Three stress variables remained in the final model: stress related to the fetus (adjusted RR = 2.6, *p*<0.001), stress related to severe marital conflicts (adjusted RR = 2.4, *p*<0.001) and stress related to serious difficulties at work (adjusted RR = 1.6, *p* = 0.031). A previous psychiatric history was also associated with prenatal depression, after adjustment for the other factors (adjusted RR = 1.8, *p* = 0.014).

**Table 7:** Adjusted relative risks of prenatal depression for variables of the final model* (*n* = 643).

Variables in the final model		Adj. RR	(95% CI)	*p*
Level of education	Primary	3.7	(1.7–8.4)	
	Secondary	2.7	(1.4–5.2)	<0.001
	Vocational qualification	1.3	(0.65–2.7)	
	Higher education	1		
History of child with major/minor birth defect	No	1		
	Yes	2.0	(1.04–4.0)	0.038
Stress related to the fetus	No	1		
	Yes	2.6	(1.6–4.1)	<0.001
Stress related to serious difficulties at work	No	1		
	Yes	1.6	(1.04–2.4)	0.031
Stress related to severe marital conflicts	No	1		
	Yes	2.4	(1.5–3.9)	<0.001
Psychiatric or psychological history	No	1		
	Yes	1.8	(1.1–2.8)	0.014

*Model resulting from a multiple forward analysis on the variables in Tables 4, 5 and 6.

## Discussion

### Principal Findings

Our study confirmed that most of the risk factors for prenatal depression are similar to those for the depression that may occur at any period in a woman's life. They include socioeconomic circumstances (as reflected by employment status and education level), personal vulnerability (as reflected by past psychiatric history and childhood adversity), and two current stressors (conflict with partner and work-related stress). However, we also found that a risk factor specific to that period, namely stress related to the fetus, was significantly associated with prenatal depression, with a rather high relative risk. A previous delivery of a child with a major or minor birth defect, was found to be a risk factor as well, though not previous pregnancy losses.

### Originality and Strengths of the Study

To our knowledge, this is the first study to have assessed simultaneously most of the major domains of risk factors for general depression identified in unified models of depression [37], [38] and specific risks for depression during pregnancy, based on data about the course of the pregnancy. Some of the risk factors we considered have seldom, if ever, been assessed in the literature in the context of prenatal depression: history of a previous child with a birth defect, specific factors related to childhood adversity (e.g., parental rejection or family secrets) and two stress factors (stress related to work or to the fetus).

The only specific form of childhood adversity that has previously been assessed in pregnant women is abuse during childhood, with significant associations evidenced among disadvantaged populations [5], [45]. We failed to find any association with physical or sexual abuse, but did find one with parental rejection and family secrets (in univariate analyses), the latter hinting at a hidden family trauma and a potential source of distress during childhood [46]–[48]. These results mirror those found in the general population. Dysfunctional parental relationships, emotional abuse and rejection have all been shown to be associated with nonmelancholic depression [49], while severe physical or sexual abuse is associated with melancholic depression [50], which is relatively infrequent during pregnancy. No cases of the latter were reported during the close medical follow-up of our population. The fact that none of the factors related to childhood adversity remained in the final model and the possible effect of a confounding variable (past psychiatric history) led us to assess an alternative model without past psychiatric history. A trend toward an association was found for family secrets (adjusted RR = 1.5, 95% CI: 0.94;2.4, *p* = 0.09).

We are not aware of any previous results concerning work-related stress. In most studies, stress is assessed by calculating the total number of stressful life events experienced by the woman within a given period of time prior to pregnancy. Significant associations have been detected in several studies [5], [29], [32], [51], but only a specific assessment of the different types of life event, such as the one we undertook here, can yield a psychologically comprehensive picture of the occurrence of prenatal depression and lead to the provision of individually tailored support plans. The amount of stress generated by an event has been found to depend on the type of coping strategy adopted by the individual and the context of its occurrence [52]. However, assessing the “contextual threat” requires lengthy semistructured interviews and complex ratings, which limits its use for large cohorts of participants. As a result, it has never been undertaken in studies of prenatal depression. We chose instead to focus on the rehearsal frequency of negative events, which is a simple measure of the event's significance to the individual and the likelihood of its affecting his or her mind. The frequency of voluntary and involuntary retrieval of an autobiographical event has been shown to be associated with the emotional intensity and centrality of that event to the person's life story [53].

We found a strong association between depression and stress related to the health of the fetus in the current pregnancy. We are only aware of one other study where an association with the pregnant mother's perception of “having complications” has been detected, albeit without any objective assessment of that risk [54]. In our study, we deemed stress related to the fetus to be present when the pregnant woman had been informed by the medical team of an ongoing risk or an uncertainty about the health of the fetus and had therefore been referred for a specific consultation or investigation. Uncertainty about the outcome of the pregnancy is a key concern in mothers-to-be and any event that is liable to trigger a serious doubt about the health or integrity of the fetus may act as a potential stress factor [55], [56].

The population we assessed was socially diverse, compared with the majority of studies, which generally focus on socially deprived women. Questionnaire acceptability was high and the percentage of nonrespondents low, compared with other studies. Depressive symptoms were assessed by means of the EPDS, a self-report instrument validated during pregnancy, using a cut-off value of 14 to ensure high specificity and sensitivity for the detection of major depression [41].

### Principal Results in Agreement with the Literature

The prevalence rate of depression during pregnancy was 11.5% in our study, which is close to the results of a major review on the subject [57]. Recent studies of the risk factors for prenatal depression have nevertheless exhibited considerable variability, with rates ranging from 6.1% [29] to 44% [34]. Variations in the characteristics of the studied population account for most of the variability, with higher rates associated with the choice of a particularly deprived population [31], [34], [45]. Conversely, in countries where pregnant women benefit from integrated support networks with easy service access, as is the case in France, depression rates are generally lower [58]. The method for assessing depression is another source of variability. As standardized interviews are difficult to implement with large samples, most previous studies have been based on self-report assessments, but with considerable diversity in the choice of instrument.

A low level of education, past psychiatric history and stress related to severe conflict with the partner were all strongly associated with prenatal depression, a result which is consistently reported in the literature. The fact that smoking, parity and low social support were only associated with prenatal depression in the univariate analysis is also consistent with most previous studies. Age was not associated with prenatal depression, which is consistent with most studies excluding pregnant girls under 17 years old, who are known to be at a higher risk of depression, particularly in deprived populations. Finally, we did not find that pregnant women with chronic medical conditions had a greater risk of depression, confirming a previous finding [59]. These results contrast with studies in the general population, in which depression has been shown to be more common in chronic disease groups [60].

### Contrasted Results

We only found a trend toward an association between socioeconomic status and prenatal depression, which could be explained by a lack of statistical power. Results about economic conditions are, however, discrepant in studies of prenatal depression. A low socioeconomic status, assessed on the basis of personal or family income, has been linked to prenatal depression after multivariate analysis in two studies [5], [61], but not in two others [54], [62].

We found no associations with ethnicity or being single. Membership of cultural or ethnic minorities has been identified as a major risk factor in several studies: not being white in Australia [45] or in the United States [34], [63], [64], and not being a native Swedish speaker in Sweden [1], [32]. Our ethnicity assessment was, however, quite indirect, as French legislation does not allow the collection of such information: we therefore had to rely on geographical origin. Being single has frequently been found to be associated with prenatal depression [1], [30]–[32], [34], [54], [63], [64] but not always [5], [27], [29]. No clear reason can be identified to explain these discrepancies. Bilszta et al. [65] postulated that a previous history of depression and poor relations with partners, rather than single mother status, are significant risk factors for elevated EPDS scores during pregnancy. However, we found no association with single mother status even in the univariate analysis.

We failed to find any association with a previous pregnancy loss in our population, which is consistent with three other studies [5], [29], [54]. However, increases in depressive symptoms have been observed after a previous abortion or miscarriage in two other studies [1], [63]. These discrepancies can partly be explained by the results of general population studies showing that the risk of developing depression is only slightly increased in the case of previous pregnancy losses [66] and weakens over time [67]. The interval of time between the previous pregnancy loss and the depression assessment has never been taken into account in studies of prenatal depression.

### Weaknesses of the Study

We were unable to use a validated instrument to assess childhood adversity for two reasons. First, the only validated scale with high criterion-related validity at the time of our study was the 70-item Childhood Trauma Questionnaire [68], but it had not yet been validated in French. Second, the use of a semistructured interview, such as the Childhood Experience of Care and Abuse interview [69], was not feasible because of the size of our cohort. We chose instead to develop a short, basic self-administered questionnaire with binary answers (yes/no) to simple questions relating to the adverse events that are most frequently mentioned, in our experience of delivering psychotherapy during the perinatal period. All these events are cited in the literature on depression. This solution had obvious limitations, but shortened the assessment's duration and improved its acceptability.

Our sample size was relatively small, compared with other studies. Neither genetic background nor biological susceptibility was explored. Moreover, we did not assess personality traits (i.e., pessimistic self-preoccupation, neuroticism or low self-esteem), even though they have consistently been found to be strongly associated with depression, in the general population as well as in pregnant women [5], [25], [29]. These explorations are particularly vulnerable to retrospective bias, since a depressed mood is, by definition, usually associated with lower self-esteem, anxiety symptoms and pessimistic self-preoccupation. Hence, interpretations of such associations in a cross-sectional study would have been extremely difficult.

### Conclusion

Our study identified several risk factors for prenatal depression which could easily be assessed in clinical practice, such as level of education, past psychiatric history and stress related to the health of the fetus. Our study also drew attention to the role of a previous delivery of a child with a major or minor birth defect, although this needs to be confirmed in a larger study. In our opinion, the role of childhood adversity also warrants further study. However, only a large prospective study of a population of nonchildbearing women will bring greater understanding of the specific role of pregnancy in the occurrence of depression and fully validate the developmental model of depression in cases of prenatal depression.
